# Deep-sea whale fall fauna from the Atlantic resembles that of the Pacific Ocean

**DOI:** 10.1038/srep22139

**Published:** 2016-02-24

**Authors:** Paulo Y. G. Sumida, Joan M. Alfaro-Lucas, Mauricio Shimabukuro, Hiroshi Kitazato, Jose A. A. Perez, Abilio Soares-Gomes, Takashi Toyofuku, Andre O. S. Lima, Koichi Ara, Yoshihiro Fujiwara

**Affiliations:** 1 Instituto Oceanográfico, Universidade de São Paulo, Praça do Oceanográfico, 191, CEP 05508-120, São Paulo-SP, Brazil; 2 Japan Agency for Marine-Earth Science and Technology (JAMSTEC), 2–15 Natsushima-cho, Yokosuka, Kanagawa 237-0061, Japan; 3 Centro de Ciências Tecnológicas da Terra e do Mar (CTTMAR), Universidade do Vale do Itajaí, Rua Uruguai, 458, P.O. Box 360, CEP 88302-202, Itajaí-SC, Brazil; 4 Departamento de Ecologia Marinha, Universidade Federal Fluminense, P.O. Box 100.644, CEP 24001-970, Niterói-RJ, Brazil; 5 College of Bioresource Sciences, Nihon University, Kameino 1866, Fujisawa, Kanagawa 252-0880, Japan

## Abstract

Whale carcasses create remarkable habitats in the deep-sea by producing concentrated sources of organic matter for a food-deprived biota as well as places of evolutionary novelty and biodiversity. Although many of the faunal patterns on whale falls have already been described, the biogeography of these communities is still poorly known especially from basins other than the NE Pacific Ocean. The present work describes the community composition of the deepest natural whale carcass described to date found at 4204 m depth on Southwest Atlantic Ocean with manned submersible *Shinkai 6500*. This is the first record of a natural whale fall in the deep Atlantic Ocean. The skeleton belonged to an Antarctic Minke whale composed of only nine caudal vertebrae, whose degradation state suggests it was on the bottom for 5–10 years. The fauna consisted mainly of galatheid crabs, a new species of the snail *Rubyspira* and polychaete worms, including a new *Osedax* species. Most of the 41 species found in the carcass are new to science, with several genera shared with NE Pacific whale falls and vent and seep ecosystems. This similarity suggests the whale-fall fauna is widespread and has dispersed in a stepping stone fashion, deeply influencing its evolutionary history.

Whale carcasses are considered the largest organic inputs reaching the deep ocean floor in a single event. Carcasses attract a suite of opportunistic and specialist organisms (see^1^ for a review) that feast on the flesh and lipid-rich bones. Specialized organisms have been evolving in these habitats for millions of years since the appearance of large ocean-going whales and other vertebrates before them^1^,^2^,^3^,^4^,^5^,^6^,^7^. Whale falls can thus be considered as sources of evolutionary novelty and biodiversity in the deep-sea, since they form isles of organic enrichment and biodiversity in an extremely food-limited environment^1^,^8^.

The degradation process of a whale carcass can pass through several overlapping successional stages^1^,^9^,^10^,^11^,^12^. During the first stages, necrophages/scavengers remove soft tissues while high densities of opportunists colonize both bones and surrounding sediments. The “sulfophilic stage” occurs when anaerobic microbial degradation of organic-enriched sediments and the lipid-rich skeleton create high fluxes of reduced compounds, which allow the development of a chemosynthesis-based community^11^,^12^,^13^,^14^,^15^. This stage shows faunal overlap with other deep-sea chemosynthetic communities, such as hydrothermal vents, cold seeps and wood falls^1^,^10^,^12^,^16^,^17^,^18^,^19^,^20^.

Based on this faunal overlap, Smith *et al*.^16^ theorized that whale falls may act as stepping-stones for faunal dispersal among different chemosynthetic communities, and could contribute to the colonization of new habitats separated by hundreds of kilometers (e.g. hydrothermal vents). In addition, this theory has also deep evolutionary implications. For instance, some of the most abundant symbiont-bearing invertebrates, such as mytilid mussels, evolved from shallow waters probably using organic-fall islands as dispersal stepping stones^1^,^21^,^22^,^23^,^24^,^25^,^26^,^27^.

Despite the importance of evolutionary and ecological relationships among biological communities at different deep-sea chemosynthetic habitats, the biodiversity and biogeography of hydrothermal vents and cold seeps has been by far much more studied. Whale falls are likely to occur worldwide along whale migratory routes as well as in whale breeding and feeding areas^16^,^20^. However, only 7 natural whale carcasses have been studied in detail in the deep-sea since 1989^16^,^20^,^28^,^29^,^30^,^31^ (although many more have been observed or remotely sampled^1^). In consequence, the advancement in the understanding of these poorly known communities has been mainly due to time-series studies of artificially implanted whale carcasses on the seafloor^1^,^20^,^32^,^33^,^34^,^35^,^36^.

Most natural and implanted deep-sea whale-fall community studies are from the deep Northeast Pacific Ocean, specifically from the California slope and Monterey Canyon^20^,^29^,^30^. Consequently, the paucity of studies on whale falls as well as the scarce data available beyond the Northeast Pacific make biogeographic and evolutionary syntheses of both whale-fall fauna, and other related chemosynthetic communities, challenging^1^.

Here we describe the community composition of the first whale carcass found in the deep Atlantic Ocean (off the S-SE Brazilian continental margin). We show that, although separated by thousands of kilometers, this abyssal Southwest Atlantic whale fall is inhabited by many lineages previously only found in the Pacific chemosynthesis-based communities. In addition, many other chemosynthetically-related genera have their bathymetric and latitudinal ranges expanded. The findings reported here have deep implications for the poorly known biogeography of deep-sea whale-fall communities and suggest a worldwide distribution for some whale-fall specialists.

## Results

### Physico-chemical characteristics of the study site and whale carcass description

**Figure 1:**
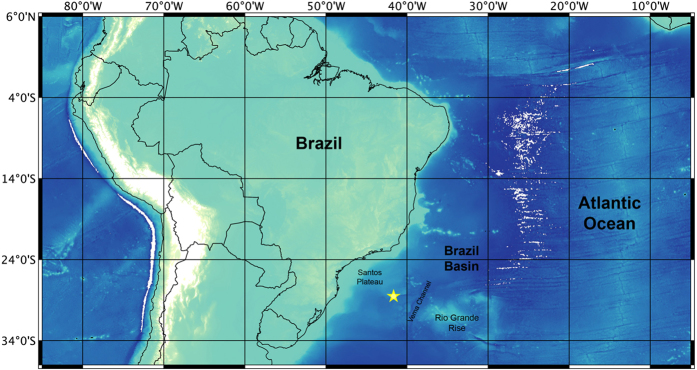
Location of the whale carcass found at the base of São Paulo Ridge at 4204 m depth. The map was created using the QGIS software, bathymetric data from CleanTOPO2 (http://www.shadedrelief.com/cleantopo2/index.html) and Word borders from Thematic Mapping (http://thematicmapping.org/downloads/world_borders.php). QGIS Development Team, 2015. QGIS Geographic Information System. Open Source Geospatial Foundation Project. http://qgis.osgeo.org. The World Borders Dataset and the data obtained from the QGIS Open Source Geospatial Foundation Project is licensed under the Attribution-Share-Alike 3.0 Unported license. The license terms can be found on the following link: http://creativecommons.org/licenses/by-sa/3.0/.

The whale fall was located ca. 700 km from the Brazilian coast at the base of the São Paulo Ridge (SPR; 28° 31.1191′ S, 41° 39.4097′ W) at a depth of 4204 m (Fig. 1). The surrounding area was characterized by a thin layer (<20 cm) of sediments overlying basaltic rocks. During our study, the area was under the influence of the Antarctic Bottom Water (AABW)^37^ with a temperature of 0.4 °C and salinity 34.7.

**Figure 2:**
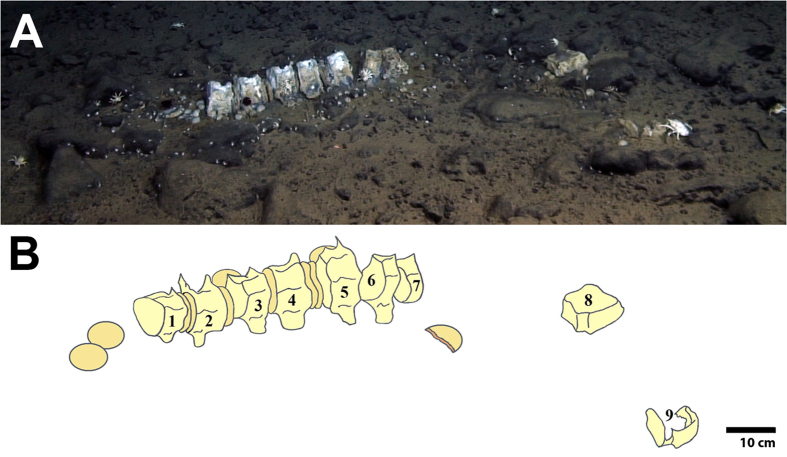
Partial Antarctic Minke whale skeleton (*Balaenoptera bonaerensis*) found at 4204 m in the Southwest Atlantic Ocean using the manned submersible *Shinkai 6500*. (**A**) Caudal vertebrae lying on a thin layer of fine sediment over basaltic rocks; (**B**) Schematic view of the whale skeleton reconstructed from *Shinkai 6500* videos. The nine vertebrae are numbered and shown in pale yellow color, while the round intervertebral discs are darker. Vertebrae were numbered from the posterior end of the animal towards the head.

Mitochondrial COI analysis revealed that the carcass belonged to an Antarctic Minke whale (*Balaenoptera bonaerensis*) (99% identity). The sequence was deposited in the DNA Databank of Japan (DDBJ) under the accession number LC106302. This partial carcass was composed of nine small vertebrae, seven of which were standing side by side. Among those, five vertebrae were loosely joined by intervertebral discs (vertebrae 1–5) (Fig. 2). Additionally, five intervertebral discs were scattered around the skeleton. No soft tissues were present on the bones, which were all exposed to the surrounding water (i.e. not covered by sediment). All vertebrae were similar in shape and dimensions (ca. 11.5 cm in diameter) and their anatomical characteristics suggest they belong to the caudal portion of the animal. The sediment underneath bones and discs was dark in color suggesting anoxia.

### Qualitative and quantitative analysis of the macrofauna assemblage and species distributions

Only epifaunal organisms larger than ca. 5 mm could be identified and counted in videos. Five phyla were recovered from the study area comprising at least 41 species (Table 1). Nematoda occurred in large numbers both inside bones and in the surrounding sediments and may be represented by more than one species. Nematodes are currently being quantified and will be treated in detail in a later publication.

**Figure 3:**
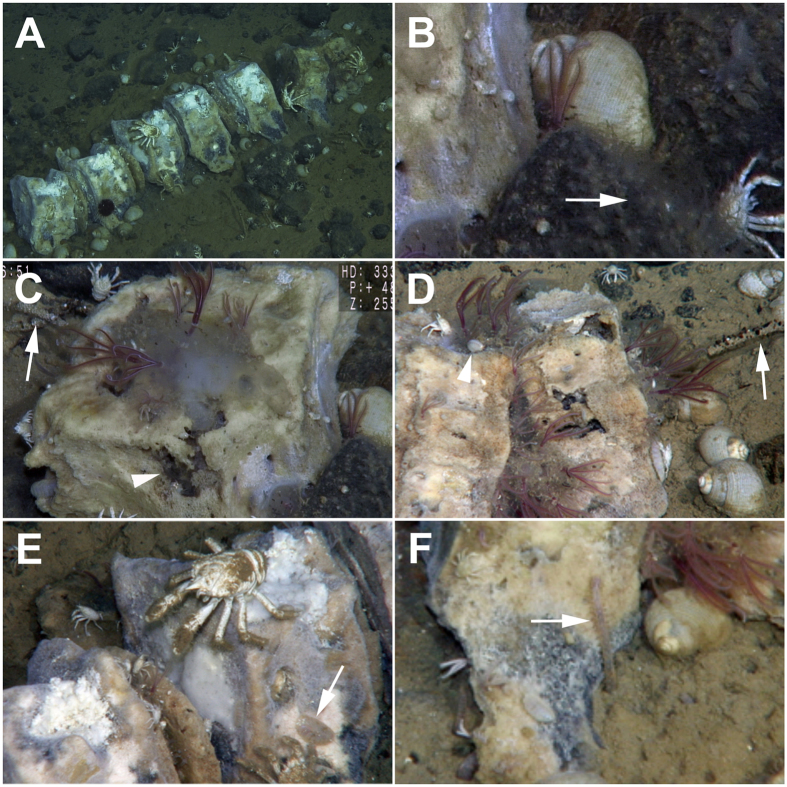
Distribution of epifauna on the whale fall and surrounding sediments and rocks. **A**) General view of the SW Atlantic whale carcass vertebrae 1–7. Note the abundant fauna and the bacterial mats on vertebrae 1–5. A dark echinoid (Echinoidea sp. 1) can be seen on top of vertebra 2; (**B**) Black basaltic rocks around the whale fall were heavily colonized by dense carpets of anemones (arrow) (up to 10 ind. cm^−2^). The large gastropod *Rubyspira* sp. nov. lies behind the red palps of *Osedax* sp. nov. Note also the small unidentified gastropods attached to the bone; (**C**) Red palps and gelatinous tubes of several *Osedax* sp. nov. in vertebra 8. Note the ampharetid polychaete tubes (arrow) and the bone degraded area (arrowhead); (**D**) Clusters of *Osedax* sp. nov. in vertebrae 6 and 7. On the surrounding sediment, *Rubyspira* sp. nov. and a tube of an ampharetid polychaete (arrow). The small lysianassoid amphipod *Stephonix* sp. lies on the top of the bone (arrowhead); (**E**) Dense bacterial mats covering vertebrae 2 and 3. Here we can see the small and the large *Munidopsis* and a polynoid polychaete (arrow); (**F**) The eyeless nereid polychaete *Neanthes* sp. nov. climbing the surface of vertebra 6 (arrow).

**Figure 4:**
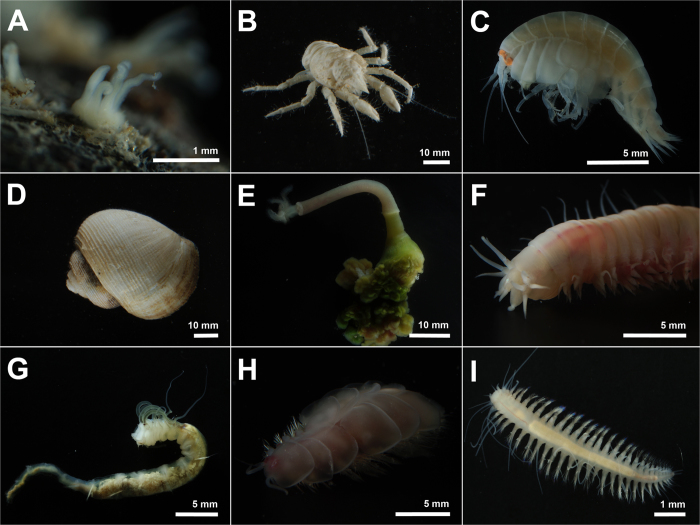
Some of the most abundant organisms collected at the 4204 m depth whale fall in the São Paulo Ridge, Southwest Atlantic Ocean. (**A**) Unidentified sea anemone inhabiting the rocks surrounding the whale skeleton; (**B**) Large *Munidopsis* sp.; (**C**) The amphipod *Stephonix* sp.; (**D**) *Rubyspira* sp. nov.; (**E**) *Osedax* sp. nov.; (**F**) *Neanthes* sp. nov.; (**G**) cf. *Grassleia* sp.; (**H**) *Bathykurila* cf. *guaymasensis*; (**I**) *Vrijenhoekia* sp. nov.

Polychaetes were the most speciose taxon on both whale bones and soft sediments, with at least 28 species (≈68%), most of which are probably new to science. Among these was a new species of the bone-eating worm *Osedax* (Fig. 3C,D). We found at least eight morphotypes of the dorvilleid *Ophryotrocha* and the new species *Capitella iatapiuna*^38^ boring into the bones, with the latter also found inhabiting the surrounding sediment sampled with a slurp gun. Three species of polynoid polychaetes, indistinguishable in video analyses, occurred on the surface of bones and sediments (Figs 3E and 4H), with a higher abundance on the former. Interestingly, antagonistic behavior could be observed in videos, where two polynoids were fighting, possibly for space or food resources (see supplemental video material). Five species of Hesionidae (*Hesiocaeca* sp. nov., *Microphthalmus* sp. nov., *Pleijelius* sp. nov. 1 and 2 and *Vrijenhoekia* sp. nov.) (Fig. 4I) and two species of cirratulids (*Raphidrilus* and *Tharyx*) were also present in both sediment and bone, except for both species of *Pleijelius* which were found only on bones. Another important species occurring in bones was the chrysopetalid *Vigtorniella*.

Some polychaetes were found exclusively in sediments surrounding the bones. Among them, one species of Ampharetidae resembling the genus *Grassleia* (Figs 3C,D and 4G) and a new species of eyeless nereid from the genus *Neanthes* were abundant (Figs 3F and 4F). Ampharetids dwelt in tubes that were widespread in sediments close to the bones and were only less abundant in videos than *Osedax* sp. nov. (Table 1) (Fig. 3C,D). *Neanthes* sp. nov. could not be counted in videos, however it was observed in videos in the anoxic sediment under bones and intervertebral discs coming out the sediment and climbing the bones, without totally leaving its gallery of burrows (Fig. 3F) (supplemental video material).

Among mollusks, a new species of the abyssochrysoid gastropod *Rubyspira* was present in large numbers (Figs 3B,D,F and 4D) and individuals were quite large in size, attaining up to 3–4 cm in length. The other gastropod was a small species found on the surface of bones (Fig. 3B). Preliminary molecular data place this small gastropod in the family Raphitomidae (Conoidea). Around the skeleton we also found many large empty shells of *Rubyspira*. No empty shells of the small unidentified gastropod were registered.

Seven species of crustaceans occurred on bones and surrounding sediments (Table 1), including a species of copepod parasitic on *Osedax*. *Munidopsis* spp. were found in large numbers being widely distributed up to 1 m away from the carcass (Figs 2, 3E, and 4B). We found two morphotypes of *Munidopsis*, one large and one small, which probably represent different species. A total of 295 individuals of both species were counted in videos (Table 1) and observations suggest these organisms feed on bacterial mats (supplemental video material). However, some of the galatheid crabs were also seen processing sediments in their mouthparts. One ovigerous female of the large morphotype was collected, which suggests that at least one of the galatheid species is reproducing on site. The amphipod *Stephonix* sp. (Figs 3D and 4C) occurred mainly on bones, frequently coming out of the bones or entering into cracks and holes in degraded areas of bones probably produced by *Osedax* activity (supplemental video material).

A small species of anemone (polyps ca. 1–2 mm in size) was observed forming extensive carpets of thousands of polyps on rocks around the carcass (Figs 3B and 4A). It was probably the most abundant epifaunal organism, with photographs suggesting a density of ca. 10 ind. cm^−2^. However, these anemones could not be counted since they could not be resolved in video analyses due to their small size. This anemone was not observed on rocks far from bones.

## Discussion

We find a close affinity between the SW Atlantic whale fall fauna with that of the NE Pacific, especially with genera found in the Monterey Canyon and off southern California^9^,^12^,^36^. We also found a large generic overlap with other chemosynthetic ecosystems. These findings have deep implications for the almost unknown biogeography of whale-fall communities and contrast/conform with patterns proposed for other chemosynthetic communities, such as vents and seeps.

Vent fields can be ephemeral and separated by large distances, occurring mainly along active mid-oceanic ridges and back arc spreading centers (reviewed in^39^). They show great endemicity and different biogeographic provinces fit well with different ocean basins and their history of geological events (reviewed in^39^,^40^). On the other hand, cold seeps may be longer lasting and widespread along all continental margins (e.g.^41^). These environments, however, do not present such endemicity and other factors such as depth rather than geography may better explain their faunal distributions (e.g.^42^,^43^).

Here we show for the first time an inter-basin distribution for many whale-fall specialists and other genera previously only known to occur in other chemosynthetic-based ecosystems (i.e., vents, seeps and wood parcels). Strikingly, some of the fauna found in the present study appears to be related to that of the NE Pacific. Five genera or 12% of all genera reported in this study were previously found exclusively in the NE Pacific (see Table 2). For instance, specialists such as the gastropod *Rubyspira* sp. nov., the polynoids *Bathyfauvelia* sp. and *Bathykurila* cf. *guaymasensis*, the ampharetid *Grassleia* sp. and the hesionid *Vrijenhoekia* sp. nov. comprise genera with distribution hitherto restricted to the Pacific^44^,^45^,^46^,^47^. In fact, for *Rubyspira* and *Vrijenhoekia* this is the first record anywhere outside Monterey Canyon and expands their bathymetric ranges by more than 1300 m depth (see^44^,^47^).

There was also a substantial overlapping with vent and seep fauna, such as *Grassleia*, a NE Pacific vents and seep inhabitant^45^, and *Bathykurila* cf. *guaymasensis*, that occurs in NE Pacific vents and whale falls^46^. The polynoid polychaete genus *Bathyfauvelia* is also registered for the first time on a chemosynthethic-related habitat. Other genera present in our study area were also found in cognate communities of the Atlantic Ocean. This is the case of the new hesionid polychaetes *Hesiocaeca* sp. nov. (sensu^48^) and *Pleijelius* sp. nov. 1 and 2, and the spionid *Lindaspio* sp. nov., previously registered in NW Atlantic methane hydrates^48^, NW Atlantic wood-fall experiments^49^ and SE Atlantic oil fields^50^, respectively (Table 2).

These findings support the stepping-stone hypothesis of Smith *et al*.^16^ and suggest that dispersal rather than vicariance is a major driver for diversification in whale fall ecosystems (see^1^,^51^). In fact, whale falls are likely to occur worldwide although heterogeneously distributed^11^. Some large baleen whales, such as humpbacks, migrate from high-latitude high-productivity feeding areas to low-latitude low-productivity breeding areas along continental margins in all oceans and to some specific oceanic islands (e.g. Hawaii) (reviewed in^52^). In addition, other species such as sperm whales, exhibit cosmopolitan distribution and can potentially sink everywhere in the ocean, especially supplying the deep ocean at equatorial latitudes^1^. In some areas carcasses may be relatively close to each other, e.g., Smith *et al*.^11^ estimated average nearest neighbor distances for whale falls from gray whales to occur every 5–16 km in the NE Pacific Ocean.

Some genera of the whale fall specialists appear to have a worldwide distribution, such as the bone-eating genus *Osedax*. *Osedax rubiplumus* illustrates well this idea having been reported in both sides of the Pacific Ocean and even in the Southern Ocean, which suggests a large inter-basin connectivity^1^. Furthermore, the present *Osedax* phylogeny does not seem to fit any specific geographical or bathymetrical pattern suggesting that dispersion is an important factor for the evolution of *Osedax* species^51^,^53^. Mitochondrial COI data (DDBJ accession number LC106303) from the new *Osedax* found in the present work place it near the NE Pacific species *O. frankpressi* (species description currently in prep.). It is the deepest *Osedax* species found to date, extending the genus depth range by more than 1300 m^28^,^54^ and it is the first found in the deep Atlantic Ocean. Similarly, mtCOI studies (DDBJ accession number LC106304) cluster *Rubyspira* sp. nov. with the two previously described species (*R. osteovora* and *R. goffrediae*^44^), both of them from the Monterey Canyon (NE Pacific). The occurrence of other Pacific genera in our study, such as *Vrijenhoekia* and *Bathykurila* cf. *guaymasensis*, also supports the idea that many whale-fall specialist lineages may be distributed worldwide (Table 2).

Thus, it is feasible to imagine a “worldwide whale-fall corridor” along continental margins, somewhat similar to the distribution of cold-seeps, but also along equatorial areas. Both “corridors” would allow faunal dispersion by a stepping-stone dispersal mechanism. This mechanism may be important for some chemosynthetic-generalist invertebrates with worldwide distributions, such as vesicomyid clams^16^,^55^, and also for whale-fall specialists.

## Methods

A whale carcass was serendipitously found at 4204 m depth in the Southwest Atlantic Ocean during a *Shinkai 6500* dive in April 24, 2013. This finding is a result of the Iatá-Piúna Research Consortium, a collaborative scientific partnership between Brazil and Japan. The Iatá-Piúna research cruise comprised two legs of the around-the-world Project Quelle 2013 (Quest for the Limit of Life) of the Japan Agency for Marine-Earth Science and Technology (JAMSTEC) using *R/V Yokosuka*.

Video surveys and sampling were carried out during two dives of the deep-sea manned submersible *Shinkai 6500* (Dives 1334 and 1336). On each dive, a detailed video survey was made, including whole community surveys and close-ups of the fauna. Owing to the small habitat size, epifaunal organisms larger that ca. 5 mm were identified to the lowest taxonomic rank possible and quantified in videos. Videos were also used to verify faunal distribution patterns along the skeleton. Images were processed using the computer program Image J^56^.

Whalebones were collected using the submersible manipulators and the fauna surrounding the area was retrieved using a slurp gun and maintained in local cold seawater during submersible ascent. Upon arrival on deck, bones were immediately transferred to a cold room at a constant temperature of 1 °C. Bone and sediment epifauna and infauna were sorted manually and under stereomicroscope. Samples were taken for morphological identification and molecular (deep-frozen at −80 °C and 99.5% non-denatured ethanol). In addition, samples were fixed in glutaraldehyde for SEM and TEM analyses.

Sediments were collected using a slurp gun and push corers and were used in the present work only for qualitative analysis. Sediment was fixed with 4% formalin (final concentration) in filtered seawater buffered with sodium tetraborate and stained with 0.05 gL^−1^. Rose Bengal dye was used to distinguish meiofauna from sediment particles. Sediment samples for metazoan meiofaunal analysis were treated according to the procedure described by^57^. The samples were washed over 63-μm mesh sieves. The sediment that remained on the 63-μm mesh sieve was resuspended and centrifuged for 10 min at 800 g with colloidal silica (Ludox HS40; Sigma-Aldrich, St Louis, MO, USA) to separate meiofauna and other lighter particles from mineral particles. The supernatants were transferred to flat-bottomed Petri dishes. Rose Bengal-stained organisms were then collected using an Irwin loop^58^, sorted into higher taxa under a binocular.

A piece of a deep-frozen vertebra was used for DNA sequencing to verify the identity of the whale skeleton. DNA was directly extracted from the bone. The vertebra sample was thoroughly washed in autoclaved and filtered seawater to eliminate surface contaminants. DNA extraction was conducted using the DNeasy Tissue Kit (Qiagen Japan, Tokyo, Japan).

The cytochrome c oxidase subunit I (COI) gene was amplified by PCR using the Ex Taq PCR Kit (Takara, Kyoto, Japan). Two oligonucleotide primers (1 μM each) and <1 μg of DNA template were added to the reaction mixtures. Thermal cycling was as follows: denatured at 96 °C for 20 s; annealed at 55 °C for 45 s; and extended at 72 °C for 2 min for a total of 35 cycles. The oligonucleotide primer sequences used for this amplification were LCO1490 and HCO2198^59^. The molecular size of the PCR products was checked with 1.2% Agarose S (Nippon Gene, Toyama, Japan) gel electrophoresis.

DNA sequencing of the amplified COI genes was performed using the BigDye Terminator Cycling Sequencing Ready Reaction Kit (PE Applied Biosystems, Foster City, CA, USA). The LCO1490 and HCO2198 primers were used in sequencing reactions. Sequencing was performed using an ABI PRISM 3100 genetic analyzer (PE Applied Biosystems).

## Additional Information

**How to cite this article**: Sumida, P. Y. G. *et al*. Deep-sea whale fall fauna from the Atlantic resembles that of the Pacific Ocean. *Sci. Rep*. **6**, 22139; doi: 10.1038/srep22139 (2016).

## Supplementary Material

Supplementary Information

Supplementary Video S1

## Figures and Tables

**Table 1:** Species collected at the SW Atlantic whale fall site at 4204 m depth.

Phylum	Class	Order	Family	Species or tag name	Number of Individuals*	Location
Cnidaria	Anthozoa	Actiniaria	n.d.	Cnidaria sp.	n.d.	Rocks
Annelida	Polychaeta	Aciculata	Dorvilleidae	*Ophryotrocha* spp.**	n.d.	Bone/Sediment
Annelida	Polychaeta	Aciculata	Nereididae	*Neanthes* sp. nov.†	n.d.	Sediment
Annelida	Polychaeta	Canalipalpata	Ampharetidae	cf. *Grassleia* sp.	40	Sediment
Annelida	Polychaeta	Canalipalpata	Chaetopteridae	*Spiochaetopterus* sp.	n.d.	Sediment
Annelida	Polychaeta	Canalipalpata	Cirratulidae	*Raphidrilus* sp.	n.d.	Bone
Annelida	Polychaeta	Canalipalpata	Cirratulidae	*Tharyx* sp.	n.d.	Sediment
Annelida	Polychaeta	Canalipalpata	Spionidae	*Lindaspio* sp. nov.	n.d.	Bone
Annelida	Polychaeta	Canalipalpata	Spionidae	*Prionospio* sp.	n.d.	Sediment
Annelida	Polychaeta	Capitellida	Capitellidae	*Capitella iatapiuna*‡	n.d.	Bone/Sediment
Annelida	Polychaeta	Phyllodocida	Hesionidae	Hesiocaeca sp. nov.∆	n.d.	Bone/Sediment
Annelida	Polychaeta	Phyllodocida	Hesionidae	*Microphthalmus* sp. nov. ∆	n.d.	Bone/Sediment
Annelida	Polychaeta	Phyllodocida	Hesionidae	*Pleijelius* sp. nov.1∆	n.d.	Bone
Annelida	Polychaeta	Phyllodocida	Hesionidae	*Pleijelius* sp. nov.2∆	n.d.	Bone
Annelida	Polychaeta	Phyllodocida	Hesionidae	*Vrijenhoekia* sp. nov. ∆	n.d.	Bone/Sediment
Annelida	Polychaeta	Phyllodocida	Polynoidae	Polynoidae	18***	Bone/sediment
Annelida	Polychaeta	Phyllodocida	Polynoidae	Polynoidae sp.	n.d.	Bone/sediment
Annelida	Polychaeta	Phyllodocida	Polynoidae	*Bathykurila* cf. *guaymasensis*	n.d.	Bone/sediment
Annelida	Polychaeta	Phyllodocida	Polynoidae	*Bathyfauvelia* sp.	n.d.	Bone/sediment
Annelida	Polychaeta	Phyllodocida	Sigalionidae	Sigalionidae	n.d.	Sediment
Annelida	Polychaeta	Phyllodocida	Sphaerodoridae	*Sphaerodoropsis* sp. nov.^☆^	n.d.	Sediment
Annelida	Polychaeta	Phyllodocida	Chrysopetalidae	*Vigtorniella* sp.	n.d.	Bone
Annelida	Polychaeta	Sabellida	Siboglinidae	*Osedax* sp. nov.^⨂^	98	Bone
Arthropoda	Malacostraca	Amphipoda	Uristidae	*Stephonyx* sp.	17	Bone/sediment
Arthropoda	Malacostraca	Isopoda	n.d.	Epicaridea sp.	n.d.	Sediment
Arthropoda	Malacostraca	Decapoda	Munidopsidae	*Munidopsis* spp.	295	Bone/sediment
Arthropoda	Maxillopoda	n.d.	n.d.	Copepoda sp.1	n.d.	Parasitic on *Osedax*
Arthropoda	Maxillopoda	Harpacticoida	n.d.	Copepoda sp.2	n.d.	Bone
Arthropoda	Maxillopoda	Cyclopoida	n.d.	Copepoda sp.3	n.d.	Bone
Nematoda	Chromadorea	Monhysterida	Xyalidae	*Theristus* sp.	n.d.	Bone
Mollusca	Bivalvia	Nuculanoida	Malletiidae	*Malletia* sp.	n.d.	Sediment
Mollusca	Gastropoda	unassigned	unassigned	*Rubyspira* sp. nov.▼	52	Sediment
Mollusca	Gastropoda	Neogastropoda	Raphitomidae	Gastropoda sp.	20	Bone epifaunal
Echinodermata	Echinoidea	indet.	indet.	Echinoidea sp.	2	Bone epifaunal
Echinodermata	Ophiuroidea	indet.	indet.	Ophiuroidea sp.	n.d.	Sediment

Each species is assigned to a location within the habitat. *Only for organisms that could be counted in videos. **Includes eight different species. ***Includes all three polynoid species collected. n.d. = not determined.

^†^Shimabukuro *et al*.*, in prep*.;

^‡^Silva *et al*.^38^.

^∆^Shimabukuro *et al*., *in prep*.;

^☆^Shimabukuro *et al*., *in prep*.;

^⨂^Fujiwara *et al*., *in prep*.;

^▾^Fujiwara *et al*., *in prep*.

**Table 2:** Genera reported in the present study and their previous geographic records.

Genus	Previously known from	Habitat	Reference
cf. *Grassleia*	NE Pacific	Hydrothermal vents and cold seeps	45
*Vrijenhoekia*	NE Pacific	Whale falls	47
*Bathykurila*	NE Pacific	Hydrothermal vents and whale falls	9,46,60
*Bathyfauvelia*	NE Pacific	Abyssal plain	61
*Rubyspira*	NE Pacific	Whale falls	44
*Hesiocaeca*	NE Pacific/NE Atlantic	Cold seeps (NE Atlantic) and whale falls (NE Pacific)	48
*Pleijelius*	NE Atlantic	Wood falls	49
*Vigtorniella*	N Pacific/NE Atlantic	Whale falls	62,63
*Lindaspio*	NE Pacific/SW Atlantic	Hydrothermal vents (NE Pacific) and oil seeps (SW Atlantic)	50
*Osedax*	All Pacific/NE Atlantic/Southern Ocean	Whale falls	1

Most genera are shared between the NE Pacific Ocean whale falls and hydrothermal vents and cold seeps.
