# Supplementary material for: Deep-sea whale fall fauna from the Atlantic resembles that of the Pacific Ocean
Source: Sci Rep. 2016 Feb 24;6:22139. doi: 10.1038/srep22139 (PMC4764926; doi:10.1038/srep22139)
Supplement: Supplementary Information [file srep22139-s1.doc]

**Deep-sea whale fall fauna from the Atlantic resembles that of the Pacific Ocean**

Paulo Y. G. Sumida, Joan M. Alfaro-Lucas, Mauricio Shimabukuro, Hiroshi Kitazato, Jose A. A. Perez, Abilio Soares-Gomes, Takashi Toyofuku, Andre O. S. Lima, Koichi Ara & Yoshihiro Fujiwara

**Video legend – Supplementary material**

**Video footage of a partial Minke whale carcass found at the base of São Paulo Ridge at 4204 m depth (SW Atlantic Ocean). Video was taking using the HD cameras of the deep manned submersible *Shinkai 6500* (Japan Agency for Marine-Earth Science and Technology – JAMSTEC). Video editing and map showed in video were created with the software iMovie 10 (Apple Inc.). Map in video shows the nearest largest city in mainland Brazil.**
